# Phenolic Compounds and Antioxidant Status of Cookies Supplemented with Apple Pomace

**DOI:** 10.3390/antiox12020324

**Published:** 2023-01-30

**Authors:** Marek Kruczek, Dorota Gumul, Anna Korus, Krzysztof Buksa, Rafał Ziobro

**Affiliations:** 1 Department of Carbohydrate Technology and Cereal Processing, Faculty of Food Technology, University of Agriculture in Krakow, ul. Balicka 122, 30-149 Kraków, Poland; 2 Department of Plant Product Technology and Nutrition Hygiene, Faculty of Food Technology, University of Agriculture in Krakow, ul. Balicka 122, 30-149 Kraków, Poland

**Keywords:** apple pomace, phenolic profile, antioxidant status, gluten-free cookies, physical features

## Abstract

The post-production leftovers after the pressing of apple juice are a rich source of health-promoting compounds, which could be used in the food industry for the manufacture of dietary foods, applicable also for people with celiac disease. This raw material is currently little used, and the cost of its disposal is considerable. Therefore, an attempt was made to enrich gluten-free cookies with different proportions of apple pomace. The content of individual polyphenols determined by the UPLC-PDA-MS/MS method, basic chemical composition, physical properties of cookies with 15%, 30%, 45%, and 60% apple pomace, were evaluated. It was found that apple pomace in gluten-free cookies caused an increase in the content of phenolic acids, quercetin derivatives, flavan-3-ols and dihydrochalcones. An elevation in protein, fat, and minerals was also observed. The growing share of apple pomace caused a significant increase in the content of total fiber, soluble, and insoluble fractions, but resulted in an increase in the hardness and darkening of the cookies while reducing their volume.

## 1. Introduction

Apples (*Malus domestica* Borkh.) are one of the most popular fruits cultivated in the world. In cultivation, they rank 4th in the world after orange, bananas, and grape [1], and their global production has increased in the last decade from 55 million tons to 81 million tons, i.e., by 38% [2,3]. Apple consumption is still growing, in 2013 reaching 9 kg/person/year and in 2017 achieving 13.2 kg/person/year [1,4]. According to statistical data, about 12% of apple pomace in Poland is treated as waste, which is associated with the costs of their disposal; the rest is used mainly in the production of pectin, which is further used in fruit processing industry, animal feed or compost [5,6]. Apple pomace is a heterogeneous mass consisting mainly of apple flesh and skin (95%), seeds (2–4%), and stalks [5,7]. These parts contain a lot of dietary fiber, so it can be considered a valuable source [8,9,10]. At the same time, apple pomace could be considered as the source of other pro-health compounds, polyphenols being an integral part of the above mentioned dietary fiber [11,12,13].

Based on the current use of apple pomace it should be clearly stated that only a small part of its applications takes into account the health-promoting nature of this plant mixture. Apple pomace is a food matrix, which can be widely used in the food industry due to valuable pro-health compounds, mainly bioactive compounds—phenolic compounds (262–999 mg/100 g d.m.), and dietary fiber (26.8–82 g/100 g d.m.) [3,12,13]. These compounds show numerous health-promoting properties, among others, a hypoglycemic effect, hypocholesterolemic, anti-carcinogenic, reducing postprandial glucose levels and arterial hypertension, have anti-inflammatory properties, antiviral, antibacterial, antiallergic, anticoagulant, and can reduce the risk occurrence of diseases such as atherosclerosis and other cardiovascular diseases, cataracts, diabetes, genetic damage, degenerative bone changes, neurodegenerative diseases including Alzheimer’s and Parkinson’s disease [14,15,16,17,18].

It should be emphasized that human cancerous changes caused by DNA damage is a growing problem in global health care. Maintaining the integrity of the genome is crucial to avoiding the initiation and progression of cancer. That is why it is so important to introduce into the diet ingredients (in this case apple pomace) containing compounds (especially polyphenols) that protect against DNA damage. In the study of Sudha et al. [19], the products with dried apple pomace showed better free radical scavenging properties, as well as cyto/DNA protective properties, which could affect the preservation of the bioactivity of these compounds after baking. The addition of dried pomace potentially increased the bioactivity of such products.

In addition, people with celiac disease typically exhibit significant oxidative stress and impaired antioxidant enzymes (superoxide dismutase (SOD), glutathione peroxidase, glutathione reductase, and catalase), which form an important antioxidant barrier in the body and are therefore susceptible to oxidant-antioxidant imbalance and DNA damage. Oxidative stress in celiac patients, measured by the level of oxidative DNA damage, can be minimized by the use of antioxidants (e.g., especially polyphenols) in the diet, which, among other things, reduce the risk of developing cancer [20,21].

In studies on patients with type 2 diabetes, a decrease in DNA damage was observed after the consumption of apple pomace extract. Among other things, type 2 diabetes is associated with increased oxidative stress and consequent oxidative DNA damage. An adequate diet rich in antioxidants can improve this impaired oxidative state [22].

According to Vazhappilly and Rupasinghe [23], apple flavonoids could prevent against oxidative DNA damage (in vitro studies) and facilitate repair mechanisms.

Apple pomace is a rich source of bioactive polyphenols and dietary fiber, while cookies usually contain a large amount of sugar and fat and small levels of dietary fiber, and therefore display limited pro-health properties. Bearing this in mind, it seems promising to use apple pomace as raw material for the production of gluten-free cookies, especially for people with celiac disease and diabetes. It would additionally broaden the portfolio of gluten-free products, which seems very important in the context of an enlarging number of people affected by celiac disease [24], who adhere to a diet based on products often deprived of nutrients, with marginal contents of pro-health compounds, as compared to traditional counterparts [25]. The trial to use apple pomace in this group of food products seems fully justified, especially as they are cheap and widespread raw materials, rich in the above-mentioned bioactive compounds.

The aim of the study was to evaluate the effect of a different share of dried apple pomace (15, 30, 45, and 60%) on the content of pro-health compounds from the group of polyphenols, and dietary fiber in gluten-free cookies. The antioxidant/antiradical potential of the cookies was characterized by three different methods: ABTS, DPPH, and FRAP. An additional aim was to evaluate the chemical composition, and important physical characteristics: texture, volume, and the color of the cookies.

## 2. Materials and Methods

### 2.1. Materials

Dried apple pomace originated from the apple concentrate production line in fruit and vegetable processing plant HORTINO Leżajsk (Poland). The material was pre-ground in a laboratory grinder (Grindomix, GM 200, Haan, Germany; two times for 5 s at 7000 rpm), and then thoroughly milled in a laboratory hammer mill Lab Mill 3100 (16,800 rpm; Perten, Sweden). Gluten-free corn flour was purchased in Bezgluten (Posądza, Poland), and the other dough ingredients (powdered sugar, margarine, eggs) at the local market.

### 2.2. Methods

#### 2.2.1. Gluten-Free Cookies Preparation

Baking of control gluten-free cookies, and the products with the share of 15%, 30%, 45%, 60% of powdered apple pomace was made according to the following procedure. All dough ingredients (Table 1), were mixed in the spiral mixer Diosna type SP 12 (Osnabrück, Germany) for about 5 min. at slow turns until the dough was kneaded. Then dough was rolled out with a rolling pin to obtain equal thickness of the dough. The cookies were cut out using one mold with a diameter of 5 cm. Baking was carried out in an electric oven MIWE CO 2 P608A (Arnstein, Germany) at the temperature of 210 °C for 12 min. After cooling to room temperature the cookies were packed in glass jars and stored for further analyses in a storage chamber at a constant temperature of 22 °C.

Chemical composition of gluten-free cookies, contents of dietary fiber and polyphenols, antiradical/antioxidant activities, color, volume, and texture were analyzed directly after baking.

Texture of the cookies were additionally assessed after 30 days of storage.

Chemical composition, dietary fiber, total polyphenol content and antioxidant/antiradical activity were also determined in the apple pomace.

#### 2.2.2. Chemical Composition

The chemical composition of the analyzed samples was determined by AOAC methods [26]. Moisture content was measured by loss on drying method (AOAC 925.10). Protein (N × 5.7) was determined by the Kjeldahl’s method (AOAC 920.87), fat by Soxhlet’s method (AOAC method no 953.38, using Soxtec Avanti 2055 (Foss, Hillerød, Denmark)) and ash (AOAC 930.05). The content of total, soluble, and insoluble dietary fiber was assessed by the method 32-07 AACCI [27]. Available carbohydrates were calculated by subtracting fat, protein, dietary fiber, and ash from 100%.

Determination of simple sugars profile after acid hydrolysis of apple pomace was done by HPLC/RI method [28]. 25 mg sample of apple pomace was treated with 2 mL of 2 M TFA (trifluoroacetic acid) at 100 °C for 3 h. After centrifugation, 1.6 mL of the clear supernatant was mixed with 3.4 mL H_2_O and neutralized with dry Na_2_CO_3_ to pH 7.20 µL was injected into an HPLC system, consisting of two columns (SP-G and SP08010, Shodex) and RI detector; the eluent was deionized water (flow rate of 0.6 mL/min); the separation was done at a column temperature of 70 °C. For calibration, monosaccharide standards at varying concentrations were applied.

Each of the above-mentioned analyses was performed in at least two replications.

#### 2.2.3. Antioxidants Content and Antioxidant/Antiradical Activity

- Extraction procedure

Antioxidants content and antiradical/antioxidant activity were determined in ethanol extracts. 0.6 g of sample was dissolved in 30 mL of 80% ethanol, shaken in the dark for 120 min. (electric shaker: type WB22, Memmert, Schwabach, Germany) and separated for 15 min. using 1050× *g*) in a centrifuge (type MPW-350, MPW MED Instruments, Warsaw, Poland). The supernatant was decanted and stored at −20 °C for further analysis.

- Total phenolic content (TPC)

Total phenolic content (TPC) was determined by a spectrophotometric method using the Folin-Ciocalteu reagent, according to Singleton et al. [29] Ether extract was 10 times diluted to a volume of 50 mL with distilled water in a volumetric flask. 5 mL of extract was combined with 0.25 mL of Folin-Ciocalteau reagent and 0.5 mL 7% Na_2_CO_3_. The contents were vortexed (WF2, Janke and Kunkel, Staufen, Germany) and stored for 30 min. in the dark place. The absorbance was measured using Helios Gamma 100–240 (Runcorn, UK), at the wavelength λ = 760 nm. The results were expressed to mg catechin or gallic acid/100 g d.m.

- Antioxidant/antiradical activity

Antiradical activity was assessed using analytical methods, namely ABTS [30]. ABTS was dissolved in the water to a 7 mM concentration. ABTS radical cation (ABTS^+•^) was produced by reacting ABTS stock solution with 2.45 mM potassium persulfate (final concentration) and allowing the mixture to stand in the dark at room temperature (12–16 h) before use. The bleaching rate of ABTS^+•^ in the presence of the sample was monitored at 734 nm using Helios Gamma 100–240 (Runcorn, UK), spectrophotometer. The ABTS^+•^ solution was diluted in PBS buffer (pH 7.4) to give absorption value of 0.700 ± 0.05 for the analysis of ethanol extracts. Volumes of 2.00 mL of ABTS^+•^ and respective ethanol extracts in PBS buffer solution were used. The ABTS^+•^ bleaching was monitored at 30 °C and the decoloration after 6 min was used as the measure of antiradical activity. Radical scavenging activity was measured as Trolox Equivalents Antioxidant Capacity (mg Trolox per g of sample’s dry mass). Trolox solutions used for calibration curve were used in the concentration range 0–2.5 mM (R^2^ = 0.9957).

- Determination of antiradical activity by synthetic DPPH radical according to Brand-Williams et al. [31]

The antioxidant activity of the ethanol extracts was measured by 2,2-diphenyl-1-picrylhydrazyl radical (DPPH). 1 mL of the extract was combined with 4 mL DPPH solution (0.012 g DPPH in 100 mL ethanol). Absorbance of sample extracts was determined with a spectrophotometer at λ = 515 nm.

Trolox (6-hydroxy-2,5,7,8-tetramethylchromane-2-carboxylic acid), designated as Tx, (R^2^ = 0.9829) was used as a standard, and the results were expressed in mg/g d.m. of Trolox equivalent (TEAC).

The FRAP method was performed according to the methodology of Oyaizu [32]. 1 mL of ethanol extract was combined with 5 mL of PBS and 5 mL of 1% K_3_(Fe(CN)_6_. A mixture of NaH_2_PO_4_·2H_2_O and Na_2_HPO_4_·2H_2_O was then added, and incubated for 20 min. in a water bath (temperature 50 °C). Then trichloroacetic acid was added. Then 5 mL was taken from the above mixture and 5 mL of H_2_O and 1 mL of 0.1 M FeCl_3_ were added. Absorbance at 700 nm was measured. The results of FRAP methodology were expressed in mg Tx/g d.m.

#### 2.2.4. Determination of Individual Polyphenols by UPLC-PDA-MS/MS

- Extraction:

Samples (about 1 g) were extracted with 10 mL of a mixture containing methanol of a purity level of HPLC (30 mL/100 mL), ascorbic acid (2.0 g/100 mL), and acetic acid in the amount of 1.0 mL/100 mL of the reagent. Extraction was carried out twice by incubation for 20 min under sonication (Sonic 6D, Polsonic, Warsaw, Poland) and mixing every 4 min. The suspension was then centrifuged at 19,000 *g* for 10 min and the supernatant was filtered through a 0.20 μm Hydrophilic PTFE membrane (marble filter Sampility Millex, Merck, Darmstadt, Germany) and used for analysis.

- Assay:

Phenolic compounds were analyzed using Aquity Ultra-Performance liquid chromatograph equipped with Binary Solvent Manager (BSM), Sample Manager (SM) combined with a PDA detector and quadrilateral time of flight (Q-TOF) Waters, Manchester, UK). The analysis was carried out on a 2.1 × 100 mm UPLC BEH C18 column containing 1.7 μm particles (Waters, Manchester, UK). The samples of the extract (0.01 mL) were eluted according to the linear gradient [33]. Anthocyanins were analyzed in the positive ion mode and the remaining polyphenols in the negative ion mode. Their identification was carried out by comparing the spectra of maximum UV absorption, molecular weight defined as mass/charge ratio, retention times, as well as fragmentation spectra with available literature data. The characteristic UV spectra were collected at the following wavelengths: λ = 320, phenolic acids; λ = 360, flavonoles; λ = 280, flavan-3-ols; λ = 340, flavones. Retention times and spectra were compared with those obtained for pure standards. Phenolic compounds were quantitatively determined using external calibration curves based on compounds selected by the analysis of standard/target structure (chemical structure or functional group). For the quantification of 3-*p*-coumaryloquinic acid, a calibration curve for *p*-coumaric acid was used in a concentration range of 0.05 to 5 mg/mL. The correlation coefficient was R^2^ ≤ 0.9998. Chlorogenic, cryptochlorogenic and neochlorogenic acids were quantified according to the internal standards in the concentration range of 0.05 to 5 mg/mL. The correlation coefficient was R^2^ ≤ 0.9998. (+) catechin, (−) epicatechin and procyanidin B_2_ were quantified according to the internal standards used in the concentration range of 0.05 to 5 mg/mL. The correlation coefficient was R^2^ ≤ 0.9998. Calibration curves for quercetin 3-*O*-rutinoside, quercetin 3-*O*-glucoside and quercetin 3-*O*-galactoside in the concentration range of 0.05 to 5 mg/mL were used to quantify quercetin derivatives. The correlation coefficient was R^2^ ≤ 0.9998. Isorhamnetin 3-*O*-rutinoside and isorhamnetin 3-*O*-glucoside calibration curves in the concentration range of 0.05 to 5 mg/mL, were used to quantify isorhamnetin derivatives. The correlation coefficient was R^2^ ≤ 0.9998. All determinations were carried out in four repetitions (*n* = 4). The results were expressed as mg/100 g d.m. Tables S1 and S2 concern the details of the assay, i.e., identification of phenolic compounds including retention time and UV-vis and mass spectral data.

#### 2.2.5. Physical Properties of Cookies with Apple Pomace

- Texture profile analysis:

Maximum force and cutting energy on the 1st and 30th day after baking have been evaluated in the texture analyzer TA.Xtplus (Stable Micro Systems, Godalming, UK) with an adapter Warner-Bratzler with a flat knife to cut the sample with speed 3 mm/s. Exponent v. 4.0.13.0 software was used. Measurements were made in seven replications, two extreme results were rejected, and the others were used to calculate the arithmetic mean. The cookies were stored in a constant temperature storage chamber (22 °C), in glass jars under dark conditions for 30 days until the next determination of texture.

- The volume of cookies:

After baking and cooling (1 h at room temperature) the volume of the cookies was analyzed with a laser measuring device Volscan Profiler 600 (Stable Micro Systems, Godalming, UK).

- Instrumental Color Analysis:

Measurement of upper surface color was carried out with the use of CM-3500d spectrophotometer (Konica Minolta Inc., Tokyo, Japan) with reference to illuminant D_65_ and a visual angle of 10°. The results were expressed using the CIE (L*a*b*) system [34] (CIE, 2004). The established color parameters were as follows: L* (lightness)—0 is black, and 100 is white; a* redness (+) greenness (−); b* yellowness (+) blueness (−). There were five replicates for each sample. Color differences (ΔE*) between samples were calculated according to the CIE formula [34]:

ΔE* = [(ΔL*)^2^ + (Δa*)^2^ + (Δb*)^2^]^½^

#### 2.2.6. Statistical Analysis

The experimental data were subjected to analysis of variance (Duncan’s test), at the confidence level of 0.05, by the use of software Statistica v. 8.0 (Statsoft, Inc., Tulsa, OK, USA). All measurements were done at least in duplicate. Pearson’s correlation coefficients were calculated in Microsoft Excel 2007 (Microsoft, Redmond, WA, USA).

## 3. Results

### 3.1. Apple Pomace Characteristics

Apple pomace is a valuable source of polyphenols, which is confirmed by the analysis performed by the UPLC-PDA-MS/MS profile of individual phenolic compounds present in apple pomace, as well as by the results of total phenolic compounds (TPC) and antioxidant potential (ABTS, DPPH, FRAP) assays (Table 2). This analysis showed that among phenolic acids, chlorogenic acid was the most abundant in apple pomace (22.7 mg/100 g d.m; Table 2). In the studies of Escarpa and Gonzalez [35] amounts of chlorogenic acid varied in the range 92–104 mg/100 g d.m. while Ćetković et al. [12] reported 3–17.6 mg/100 g d.m. The level of cryptochlorogenic acid was 20 times lower and the level of *p*-coumaroquinic acid was 13 times lower in comparison to chlorogenic acid in the analyzed pomace (Table 2). The amount of *p*-coumaroquinic acid was earlier shown to be 0.18 mg/100 g d.m. in the studies by Kammerer et al. [13]. According to Lyu et al. [1], the content of phenolic acids in apple pomace was in the range 52.3–154.2 mg/100 g d.m. Quercetin derivatives dominated among the flavonols, including quercetin-3-*O*-galactoside; quercetin-3-*O*-rhamnoside, and quercetin-3-*O*-xyloside, as well as quercetin-3-*O*-glucoside. Other research groups reported the level of quercetin-3-*O*-glucoside 28.6–61.0 mg/100 g d.m. [12] and 52.1–68.1 mg/100 g d.m. [35]. According to Lyu et al. [1], the quantity of flavonols and anthocyanins ranged between 215.3–373.4 mg/100 g d.m.

The main dihydrochalcone was phloridzin, whose quantity was 21.3 mg/100 g d.m. (Table 2). According to Leyva-Corral et al. [36], its quantity was 17.97 mg/100 g d.m., while Lyu et al. [1] determined 68–253 mg/100 g d.m.

Among the flavan-3-ols we distinguish catechin, procyanidins B2, and epicatechin (Table 2). Other authors evaluated the catechin content at the level of: 1.7–12.7 mg/100 g d.m. [12]; 0.24 mg/100 g d.m. [13]; and 0.94–1.4 mg/100 g d.m. [36]. The amount of epicatechin in apple pomace analyzed by other authors was 2.4–17.3 mg/100 g d.m. [12]; 0.93 mg/100 g d.m. [13]; 14–19 mg/100 g d.m. [37]; 12.23 mg/100 g d.m. [36]. The amount of procyanidin B2 varied appropriately in the studies of other authors: 2.3–10 mg/100 g d.m. [35]; 0.93 mg/100 g d.m. [13] and 9.3–16 mg/100 g d.m. [37]. The content of the above-mentioned compounds depends on many factors, e.g., apple variety, soil and climate conditions, agronomical techniques, juice production technology, methods of sample preparation [38].

**Figure 1:**
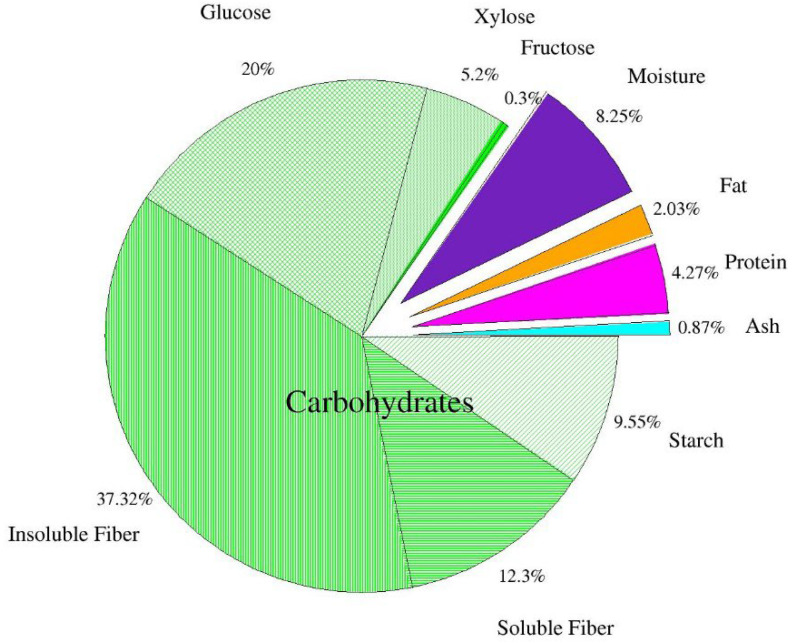
Chemical composition of dried apple pomace (*n* = 2).

Figure 1 shows the chemical composition of the dried apple pomace. It was found that dietary fiber is the largest part of dry matter in apple pomace. The results confirm earlier data of Bhushan et al. [5] and Sato et al. [39], who reported that dietary fiber of apple pomace consists of insoluble (2/3) and soluble fractions (1/3). According to the authors, the observed 1:2 ratio between soluble and insoluble fractions is beneficial from a nutritional point of view. The level of other constituents, protein, fat, carbohydrates, and ash is usually consistent with other reports on apple pomace. Pieszka et al. [40] reported the levels of protein, fat, soluble sugars, and ash to be 6.91 g/100 g d.m., 3.30 g/100 g d.m., 0.61 g/100 g d.m., 1.48 g/100 g d.m., respectively. Leyva-Corral et al. [36] determined 3.42 g/100 g protein, 0.26 g/100 g d.m. fat, 1.68 g/100 g d.m. ash. Gazalli et al. [9] obtained similar results: protein—5.11 g/100 g d.m., fat—3.12 g/100 g d.m, ash—3.97 g/100 g d.m. Lyu et al. [1] reported 3.8–4.7 protein, 0.6–4.2 fat, 42.5 total dietary fiber (including cellulose, hemicellulose, and lignin), 1.5–2.5 ash (including P, Ca, Mg, and K) and 45.1–83.8% carbohydrates. Some variation in these results may be explained by different apple varieties, climatic, agrotechnical, and soil conditions [40].

According to Waldbauer et al. [41], total sugar content equals 45.79 g/100 g d.m., including: glucose 7.51 g/100 g d.m.; fructose 15.96 g/100 g d.m.; sucrose 8.36 g/100 g d.m. According to Lyu et al. [1], the content of glucose is 22.7 g/100 g, fructose—25.6 g/100 g and galactose—6.75 g/100 g d.m.

Some inaccuracies regarding the differences in glucose, fructose, and sucrose in the analyzed apple pomace compared to the study by Waldbauer et al. [41] are most likely related to the applied acid extraction using ultra-performance liquid chromatography (HPLC). The result of the applied acid extraction was an increase in glucose content, partly due to the acid hydrolysis of starch and the breakdown of sucrose present in the apple pomace. Furthermore, it should be mentioned that the absence of fructose and sucrose in this analyzed post-production raw material is also related to the applied acid extraction during the HPLC analysis, which led to their breakdown. Kołodziejczyk et al. [42] also indicate that the variety of apples used for juice production have a strong influence on the saccharide composition of the pomace. Indeed, in the pomace analyzed by the above-mentioned research team, obtained from 28 apple varieties, the saccharide contents ranged for glucose 1.8–16.2 g/100 g d.m., fructose 10.8–30.6 g/100 g d.m., and sucrose 0.5–21.3 g/100 g d.m. According to other authors [39,43,44], the contents of sugars in apple pomace are 3.4–24 g/100 g d.m. sucrose; 2.5–12.4 g/100 g d.m. glucose and 18–31 g/100 g d.m. fructose, which was also explained by the varietal factor.

### 3.2. Characteristics of Cookies with a Different Share of Apple Pomace

**Figure 2:**
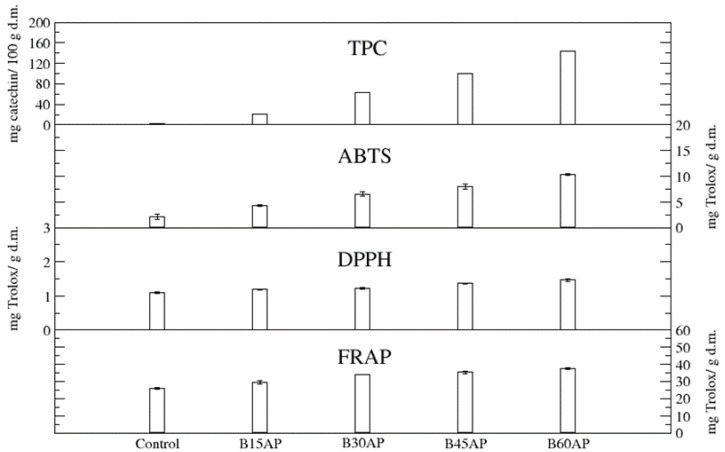
TPC and antioxidant potential of gluten-free cookies with apple pomace (*n* = 2).

The content of total polyphenols (TPC) in gluten-free cookies ranged from 20.58 to 143.16 mg of catechin/100 g d.m. in comparison to the control, which contained 3.21 mg of catechin/100 g d.m. Thus, the apple pomace used guaranteed an up to 44-fold increase in these bioactive compounds in gluten-free cookies with a share of 60% pomace relative to the control. Even the lowest proportion of apple pomace caused a 6-fold increase in polyphenols in gluten-free cookies (Figure 2).

The antioxidant potential, measured by three methods, unequivocally confirmed that the introduced polyphenolic compounds present in apple pomace provide high antiradical and antioxidant activity of cookies with the participation of the pomace (Figure 2). Moreover, it was found that the antioxidant potential, estimated by three methods, of gluten-free cookies with 15% of ground apple pomace was very significant. It can be suggested that such high antiradical and antioxidant activity, in addition to polyphenols, will be partly influenced by newly formed compounds during baking, i.e., the products of the Maillard reaction [45,46,47]. They could affect the antiradical and antioxidant activity of cookies to a greater extent than in the case of bread, due to the greater surface area of temperature exposure and the presence of a substrate suitable for the formation of Maillard reaction products i.e., sugars in the cookie baking recipe.

A strong correlation was found between the total phenolic content (TPC) of the analyzed cookies and ABTS (R^2^ = 0.99) as well as DPPH (R^2^ = 0.98).

All the methods used in this work to determine the in vitro antioxidant status (DPPH, ABTS, FRAP) refer to hydrophilic and hydrophobic polyphenols. The aforementioned methods differ in principle, as ABTS and DPPH are used to eliminate free radicals by the polyphenols in the sample, while FRAP is used to reduce iron from Fe^3+^ to Fe^2+^ to prevent the oxidation reaction. Hence, there will be differences in the values of the determined antioxidant activities [48]. Additionally important in the determination of antioxidant activity by the DPPH method, is the fact of interference from other compounds that are absorbed at the wavelength used in this determination (515 nm) [49]. This results in differences between antioxidant activity values determined by ABTS and DPPH methods in the same samples.

In a study by Mir et al. [50] on the effect of apple pomace on the quality of gluten-free rice crackers, an increase in the polyphenol content of these crackers with increasing levels of addition was also reported (from 3 to 9%). This increase ranged from 14% to 34%, compared to the control sample. According to Mir et al. [50], the increase in the aforementioned phenolic compounds was parallel to the level of additive used. Similarly, in the study presented in this work, the increase in the amount of TPC was consecutive to the increase in the proportion of apple pomace in the cookies. In addition, the explanation of the possibility of enriching cookies with bioactive compounds present in apple pomace is in line with the views of Mir et al. [50]; although baking caused losses, the apple pomace used enabled an increase in bioactive compounds that contributed to an increase in the antioxidant potential of the product. Additionally, Mir and his team [50] indicate that the contribution to the antioxidant and antiradical activity of bakery products will be the products of the Maillard reaction. In a study by Šarić et al. [51] concerning gluten-free cookies with different proportions of blueberry and raspberry pomace, a significant content of bioactive compounds was observed after the introduction of the pomace rich in these constituents. Gluten-free cookies with a mixture of the above-mentioned pomace with different proportions (total addition of 30%) were characterized by a higher content of total polyphenols by 725 times to 2500 times compared to the control. The content of anthocyanins was determined in this product in the range of 41 to 257 mg/100 g d.m. only after the use of enrichment mixtures of berry and raspberry pomace, in contrast to the control, which did not show the presence of these compounds (consisting of a mixture of corn starch, potato starch and rice flour). In addition, Šarić et al. [51] proved that cookies containing only blueberry pomace contained six times more TPC than cookies with raspberry pomace, and the anthocyanin content of these products was 1.6 times higher when using blueberry pomace than raspberry pomace. The same cause was responsible for many times higher antiradical activity of these products determined using the DPPH radical. The aforementioned authors, such as Mir et al. [50], explain this huge increase in the content of bioactive components in cookies by the use of pomace from colored fruits for the production of these products. So, we can say that the research presented in this paper confirms the views of the above-mentioned authors [50,51].

The UPLC-PDA-MS/MS method was used to analyze the qualitative and quantitative profile of phenolic compounds in gluten-free cookies with apple pomace and control. The introduction of apple pomace into the cookies resulted in the appearance of quercetin derivatives, flavan-3-ols, dihydrochalcones, and an increase in phenolic acids, especially chlorogenic acid (from 13 to 41 times) crytpochlorogenic acid (from eight to 26 times) as compared to the control (Table 3). It was the apple pomace that was a source of these compounds (Table 2 and Table 3). However, it should be noted that quercetin derivatives are thermally labile, which leads to the formation of phenolic acids (Table 3). The presence of other phenolic acids in addition to those mentioned above should be explained by the integrated action of many factors including dough mixing, and above all, the thermal decomposition of quercetin derivatives [52,53,54,55]. Taking into account the fact that the amount of quercetin derivatives in apple pomace is high (Table 2), the process of baking applied in the manufacture of cookies with apple pomace contributes to their thermal degradation and consequently to the increase of phenolic acids much higher than it would be expected from the share of pomace in this product. However, it should be noted that some of the phenolic compounds can be degraded, therefore two of them (isorhamnetin-3-*O*-glucoside and isorhamnetin-3-*O*-galactoside) did not increase despite the increase of pomace percentage in cookies.

The appearance of quercetin derivatives as well as flavan-3-ols and dihydrochalcones, or 13 times increase in chlorogenic acid and eight times increase in cryptochlorogenic acid in cookies with 15% apple pomace, as compared to the control (Table 3), is highly beneficial; despite this, some authors regard baking process to result in a loss of polyphenol compounds [53,54]. Other additions of apple pomace from 30 to 60 percent provided a higher content of quercitin derivatives and the amount of isorhamenetin derivatives did not change in the pomace supplemented cookies compared to the control. In the case of chlorogenic acid and its isomer cryptochlorogenic acid, 60% of apple pomace provided a 41-fold and 26-fold increase, respectively, in cookies with pomace compared to cookies without pomace. Of the flavan-3-ols and dihydrochalcones, cookies with 60 percent contained the highest levels of these bioactive compounds compared to the other samples.

According to the above-mentioned authors, a reduction in these compounds can reach up to 60 percent. These losses are affected by many factors such as thermal degradation, enzymatic, oxidative, isomerization/epimerization processes, and the decarboxylation of phenolic acids, most noticeably to 4-vinyl-guaiacol [52,53,54]. Additionally, the losses of these phenolic compounds could be caused by forming complexes with polysaccharides, e.g., starch [55], which were used as raw materials in cookie baking.

Even though thermal processes such as baking contribute to the loss of polyphenols, the smallest addition of apple pomace (15%), which is a powerful source of bioactive compounds, guarantees a significant amount of phenolic compound in the final products. The appearance of quercetin derivatives, flavan-3-ols, dihydrochalcones, 13-times increase in chlorogenic acid or eight times increase in cryptochlorogenic acids in such cookies, as compared to control, is very positive in terms of nutritional quality. In the context of the low nutritional value and lack of pro-health compounds in gluten-free products, it seems that apple pomace significantly enriches the cookies in pro-health compounds, especially quercetin derivatives, phenolic acids, and phloridzin, as compared to control. Quercetin reduces the risk of various types of cancer and cardiovascular diseases [14,56]. The observed increase in chlorogenic acid is also highly valuable, as it is considered as a potent antioxidant in the human diet, and has a chemopreventive role (against cancer and neurodegenerative diseases) [57]. All the analyzed products displayed an elevated level of phloridzin, which together with quercetin reduces the risk of diabetes type II [58]. Moreover, Lyu et al. [1] proved that dihydrochalcones have antidiabetic properties, promote bone-forming, and reveal some potential in treating obesity and blastogenesis.

Table 4 shows the levels of the main chemical components (moisture, protein, fat, ash, and available carbohydrates) in gluten-free cookies with different additions of apple pomace. In the case of protein, its content decreased from 7.45 g/100 g d.m. in the control sample by 6 to 22% in cookies with apple pomace (Table 4). This decrease was directly proportional to the increasing level of apple pomace in the cookies. Most likely it is related to the fact that the corn flour used to make cookies contained more protein (6.4 g/100 g d.m.) [59] compared to apple pomace (4.61 g/100 g d.m.; Figure 1). Mir et al. [50] achieved a similar result while studying the effect of apple pomace on the quality of gluten-free rice crackers, as they found a reduction in the protein content of these crackers with increasing levels of the added pomace (from 3% to 9%). According to the above-mentioned authors, the decrease to the control ranged from 7% to 12%.

Gluten-free cookies with 15% and 30% apple pomace had a level of fat close to the control. Only the higher share of apple pomace, 45% and 60% contributed to the increase of this ingredient by 4% and 8%, respectively, compared to the control (Table 4). Bearing in mind the fact that each type of cookie contained the same amount of fat added in the form of margarine (Table 1) it should be assumed that this slight increase was related to the additive used, which as mentioned earlier is also a source of fat (2.03 g/100 g d.m.; Figure 1) and contains 18% more of it than the corn flour used in the recipe [59]. Mir et al. [50] obtained similar results, as the minimum addition level of apple pomace (from 3 to 9%) did not affect the fat content of crackers.

An increase in the amount of minerals in gluten-free cookies has been observed with a share of apple pomace increasing from 14% to 58% to the control (Table 4), which was related to a large quantity (from 15% to 60%) of apple pomace, which replaced corn flour, substantially lower in minerals (0.05 g/100 g d.m.) [5]. Similar results were obtained by Mir et al. [50] in their research on the influence of apple pomace on the quality of gluten-free rice crackers, who found that with an increasing share of apple pomace (3%, 6%, 9%) the amount of mineral components increased, especially for calcium, copper, iron, potassium, and silicon. Most likely it was due to the addition, because as reported by O’Shea et al. [60], apple peel is a rich source of calcium, zinc, iron, copper, and magnesium. According to Rupasinghe et al. [61], wheat muffins with the share of ground apple peel were characterized by a higher content of minerals along with an increasing share of ground apple peel in the finished product.

Gluten-free products very often contain low amounts of dietary fiber, the proper amount of which in the diet assures proper functioning of the digestive system. An increase in the content of dietary fiber fractions was noticed from eight to 29 times for the insoluble fraction, and from 1.5 to five times for the soluble fraction in the cookies with the pomace as compared to the control (Table 4). The cookies with apple pomace had a total fiber content ranging from 8.83 to 27.71 g/100 g d.m., its content was adequate to the level of added apple pomace. The highest content of fractions of dietary fiber was provided by the highest share of apple pomace (60%) in cookies (Table 4). Such a huge increase of dietary fiber in cookies enriched with apple pomace is highly beneficial from a nutritional point of view. The insoluble fraction of dietary fiber has a preventive and therapeutic activity in the treatment of colorectal diseases (habitual constipation, irritable bowel syndrome, hemorrhoids). On the other hand, the soluble fraction has hypocholesterolemic, hypoglycemic, and antitumor activities [62]. In studies made by Mir et al. [50] regarding the influence of apple pomace on the quality of gluten-free rice crackers, an increase in the insoluble fraction, soluble fiber, and total fiber content in these crackers was found with increasing levels of the additive (from 3% to 9%). Compared to the control, this increase ranged from 19% to 59% for the insoluble fraction, from 435% to 1629% for the soluble fraction, and from 42% to 146% for the total dietary fiber.

After the introduction of apple pomace into gluten-free cookies, a reduction in available carbohydrates was observed compared to the control, because of the increased fiber content, which was added with this ingredient (Table 4).

To summarize, the application of apple pomace, which is a source of dietary fiber and polyphenols integrally bound to its fractions, results in the development of the pro-health value of cookies in comparison with control.

### 3.3. Physical Properties of Gluten-Free Cookies

The obtained values of hardness increased with the increasing share of apple pomace and with the storage time. The cookies with the highest share of apple pomace (60%) were characterized by the highest hardness after the first and thirtieth day of storage. An increase in hardness after 30 days of storage was found in the range from 3% to 20% to the hardness of the products one day after production (Table 5). The cookies with a 45% share of apple pomace were characterized by the highest increase in hardness during storage.

The same results were obtained by Sakr and Hussien [63], where the increasing proportion of sweet chickpea flour in gluten-free cookies increased their hardness. The cookies with the highest (45%) share of chickpea flour were characterized by the highest hardness—67% more compared to the control. The mentioned authors concluded that the hardness increased as a result of the introduction of fiber with chickpea flour. This was also confirmed by the research of Demirkesen [64], where the highest share of chestnut flour resulted in the hardest products. This was most likely due to the high viscosity of the dough with chestnut flour, which limited the introduction of air while mixing the ingredients and thus resulted in the high hardness of the baked, compact products. Additionally, the high sugar content of the chestnut flour could prevent gelatinization of some of the starch granules during baking. Therefore, they were unable to form a continuous structure which resulted in harder cookies [61,65]. A similar role can be played by fiber, which by binding large amounts of water limits its availability to starch, thus limiting its degree of gelatinization. Such a relationship was observed by Cervini et al. [65] studying the effect of the addition of resistant starch from sorghum on gluten-free cookies. The authors found an increase in the hardness of gluten-free cookies along with an increase in the amount of resistant starch. Similarly, Bolek [66] observed a significant increase in hardness of wheat cookies with increasing amount of olive stone powder, which he attributed to the significant dietary fiber content in this material. On the other hand, the hardness of cookies could also be influenced by the type of dietary fiber. Jia et al. [67] found that the addition of soluble dietary fiber from defatted rice bran to wheat flour cookies reduced the hardness of the cookies. The apple pomace used in the present study contained mainly insoluble fiber (Figure 1), which may have been the reason for the increase in cookie hardness with an increasing amount of this ingredient (Table 4 and Table 5). In general, texture parameters are a resultant of the composition of the matrix built up by proteins, fat, carbohydrates with embedded remnants of starch granules and their interactions during baking [65,67].

In studies by Šarić et al. [51], regarding cookies with raspberry and blueberry pomace, it was found that the raspberry pomace decreased the hardness of the obtained cookies, at the same time increasing their moisture content, while the blueberry pomace increased hardness while reducing their moisture content.

The obtained cakes with apple pomace were characterized by 4% to 6% lower volume compared to the control (Table 5). The lowest volume per 100 g was that of cookies with the largest 45% and 60% share of apple pomace, but the volume reduction was not significantly different in the case of other shares.

Color is one of the basic parameters for visual evaluation, affecting consumers’ acceptance of products. Gluten-free cookies just after baking were characterized by the parameter L* determining the brightness of the color in the range 35.74–75.86, the brightest were the control cookies. The addition of apple pomace decreased this parameter from 32% to 53% compared to the control. The cookies with the highest 60% share of apple pomace were characterized by the lowest brightness. The enrichment of the cookies with apple pomace caused an increase in the redness parameter a* from 11 to 20% compared to the control, and a decrease in the value of the yellowness parameter b* from 43 to 67% compared to the control (Table 5). The highest increase was caused by the lowest 15% (for the parameter a*) and the highest 60% (for the b* parameter) share of apple pomace, respectively. The total color difference (Δ*E*) decreased with an increasing proportion of apple pomace (13.62–11.27) compared to the control. Apple pomace significantly changed the color of the cookies obtained, which was an obvious consequence of the rising content of this component, and its natural dark color, caused by enzymatic browning during drying. Additionally, the presence of polyphenols in apple pomace could result in the Maillard reaction with sugars causing further darkening of the obtained products [68]. Similar results were obtained in the studies by Ajila et al. [69], who analyzed the effect of the addition of powdered mango peel on the properties of cookies and Mir et al. [50], where the addition of apple pomace also darkened the obtained crackers.

In the context of the low nutritional value and marginal health-promoting value of gluten-free products mentioned in the introduction, it seems that the apple pomace used definitely enriched the gluten-free products developed in this work, in health-promoting compounds from the polyphenol group and dietary fiber. The observed increase in quercetin derivatives in the analyzed gluten-free products with the participation of apple pomace positively influenced the health-promoting value of the products due to the fact that quercetin reduces the risk of various types of cancer and heart disease [14,56,58]. In addition, an increase in the amount of chlorogenic acid was observed in all analyzed gluten-free products involving pomace, in relation to the control sample. It is important especially in the context of the chemopreventive role of this phenolic acid (anticancer and factor against neurodegenerative diseases) [57]. Additionally, the increase in the level of phloridzin in all analyzed gluten-free products containing apple pomace speaks in favor of this post-production raw material, as phloridzin acts synergistically with quercetin in reducing the risk of type II diabetes [70,71,72].

In conclusion, basing on the above-mentioned results, it can be suggested that the health-promoting compounds contained in pomace, which primarily include dietary fiber and a wide range of phenolic compounds, act synergistically for the beneficial effects on the human body. Because all these compounds are localized in the food matrix, their isolation may not have such beneficial results. Therefore, the use of apple pomace as a source of health-promoting compounds for the biofortification of gluten-free products seems to be justified, also due to the fact of its easy acquisition and low price.

It can be suggested that in a further stage the research will encompass nutritional studies. Such studies are especially justified taking into account that people with celiac disease usually exhibit significant oxidative stress and impaired enzyme function, and are therefore susceptible to oxidation-antioxidant imbalance and DNA damage, resulting in consequent cancer. On the other hand, it should be noticed that the number of people on a gluten-free diet is increasing every year in the world population.

## 4. Conclusions

It was observed that apple pomace introduced phenolic acids, quercetin derivatives, flavan-3-ols, and dihydrochalkones into gluten-free cookies. The appearance of quercetin derivatives, flavan-3-ols, dihydrochalcones, a 13-times increase in chlorogenic acid or eight times increase in cryptochlorogenic acids, as compared to control, could be observed in cookies with the smallest addition of apple pomace (15%). The presence of apple pomace in cookies caused an increase in the insoluble fraction of dietary fiber (8–29 times), the soluble fraction (1.5–5 times), and total fiber (3.5–13 times), to control. In terms of the chemical composition, a decrease in protein content was observed in cookies with apple pomace, a slight increase in fat, and an increase in the content of minerals in the range from 14% to 58% compared to the control. The hardness in gluten-free cookies with apple pomace increased, compared to the control, with their increasing proportion and storage time.

Summarizing, it was found that apple pomace is a source of dietary fiber and polyphenols (especially chlorogenic acid, catechin, epicatechin, quercetin derivatives, procyanidin B2, and dihydrochalkones—phloridzin), which may be successfully applied in biscuit baking.

## Figures and Tables

**Table 1:** Ingredients of gluten-free cookies (g).

Sample	Corn Flour	Apple Pomace	Powdered Sugar	Margarine	Egg Mass
Control *	364	0	46	208	32
B 15AP	309	55	46	208	32
B 30AP	255	109	46	208	32
B 45AP	200	164	46	208	32
B 60AP	146	218	46	208	32

* Sample coding: Control—control gluten-free cookies, B 15AP—gluten-free cookies with 15% share of dried apple pomace, B 30AP—gluten-free cookies with 30% share of dried apple pomace, B 45AP—gluten-free cookies with 45% share of dried apple pomace, B 60AP—gluten-free cookies with 60% share of dried apple pomace.

**Table 2:** Quantity of phenolic compounds (mg/100 g d.m.) and antioxidant potential of apple pomace (*n* = 2).

Group of Compounds	Name	Content
Flavonols	quercetin-*O*-rutinoside	4.21 ± 0.17 ^1^
	quercetin-3-*O*-galactoside	32.63 ± 0.21
	quercetin-3-*O*-glucoside	8.40 ± 0.05
	quercetin-3-*O*-arabinoside	11.55 ± 0.03
	quercetin-3-*O*-xyloside	19.60 ± 0.00
	quercetin-3-*O*-rhamnoside	26.80 ± 0.00
	isorhamnetin-3-*O*-galactoside	1.30 ± 0.42
	isorhamnetin-3-*O*-glucoside	0.90 ± 0.00
Phenolic acids	chlorogenic acid	22.70 ± 0.23
	cryptochlorogenic acid	1.20 ± 0.07
	*p*-coumaroylquinic acid	1.83 ± 0.20
Flavan-3-ols	(+) catechin	1.67 ± 0.03
	procyanidin B2	3.79 ± 0.00
	(−)epicatechin	1.35 ± 0.23
Dihydrochalcones	phloretin-2-*O*-xylosylglucoside	1.90 ± 0.11
	phloretin 2-*O*-glucoside (phloridzin)	21.30 ± 0.08
Sum		161.13
TPC (mg gallic acid/100 g d.m.)		470.5 ± 1.5
ABTS (mg Trolox/g d.m.)		12.16 ± 0.43
DPPH (mg Trolox/g d.m.)		3.09 ± 0.20
FRAP (mg Trolox/g d.m.)		27.7 ± 0.0

^1^ Mean ± SD.

**Table 3:** Quantity of phenolic compounds (mg/100 g d.m.) in gluten-free cookies with apple pomace (*n* = 2).

Compounds	Control *	B 15AP	B 30AP	B 45AP	B 60AP
Flavonols (mg/100 g d.m.)					
isorhamnetin-3-*O*-galactoside	n.d.	0.21 ± 0.04 a	0.23 ± 0.03 a	0.25 ± 0.02 a	0.27 ± 0.02 a
isorhamnetin-3-*O*-glucoside	n.d.	0.27 ± 0.01 a	0.29 ± 0.03 a	0.28 ± 0.03 a	0.30 ± 0.02 a
quercetin-*O*-rutinoside	n.d.	0.57 ± 0.01 a	0.74 ± 0.03 b	1.16 ± 0.07 c	1.29 ± 0.02 d
quercetin -3-*O*-galactoside	n.d.	5.27 ± 0.00 a	6.75 ± 0.05 b	8.72 ± 0.00 c	10.53 ± 0.04 d
quercetin -3-*O*-glucoside	n.d.	1.28 ± 0.00 a	1.56 ± 0.08 b	2.30 ± 0.04 c	3.13 ± 0.03 d
quercetin -3-*O*-arabinoside	n.d.	2.09 ± 0.02 a	2.31 ± 0.04 b	3.04 ± 0.03 c	4.26 ± 0.07 d
quercetin -3-*O*-xyloside	n.d.	3.85 ± 0.03 a	4.00 ± 0.00 b	5.26 ± 0.02 c	6.49 ± 0.02 d
quercetin -3-*O*-rhamnoside	n.d.	4.63 ± 0.04 a	5.02 ± 0.03 b	7.35 ± 0.08 c	9.21 ± 0.06 d
luteolin 6-*C*-hexoside-*O*-hexoside	n.d.	n.d.	n.d.	n.d.	n.d.
luteolin *O-* hexoside-C-hexoside	n.d.	n.d.	n.d.	n.d.	n.d.
Phenolic acids (mg/100 g d.m.)					
chlorogenic acid	0.33 ± 0.08 a	4.34 ± 0.13 b	6.63 ± 0.09 c	10.27 ± 0.17 d	13.57 ± 0.08 e
cryptochlorogenic acid	0.03 ± 0.00 a	0.24 ± 0.02 b	0.41 ± 0.07 c	0.53 ± 0.07 c	0.78 ± 0.05 d
caffeoylquinic acid	n.d.	0.18 ± 0.03 a	0.18 ± 0.02 a	0.19 ± 0.00 a	0.18 ± 0.00 a
*p*-coumaroylquinic acid	0.01 ± 0.00 a	0.25 ± 0.00 b	0.24 ± 0.02 b	0.27 ± 0.04 b	0.28 ± 0.00 b
caffeoyl-dihydroxyphenyllactaoyl-tartaric acid	n.d.	0.56 ± 0.00 a	0.74 ± 0.15 b	0.82 ± 0.07 b	0.93 ± 0.11 b
2-*O-p* -coumaroylglicerol	n.d.	n.d.	n.d.	n.d.	n.d.
1-*O*-*p*-coumaroylglicerol	n.d.	0.14 ± 0.01 a	0.19 ± 0.03 b	0.25 ± 0.02 b	0.37 ± 0.05 c
*p*-coumaroylspermidin	0.08 ± 0.00 b	0.06 ± 0.00 a	0.03 ± 0.00 a	n.d.	n.d.
di-*p*-coumaroylspermidin	0.14 ± 0.04 b	0.10 ± 0.00 a	0.06 ± 0.00 a	0.02 ± 0.00 a	n.d.
Ferullyquinic acid	0.09 ± 0.00 a	0.17 ± 0.00 b	0.23 ± 0.07 b	0.31 ± 0.03 b	0.47 ± 0.06 c
Flavan-3-ols (mg/100 g d.m.)					
(+) catechin	n.d.	0.41 ± 0.00 a	0.42 ± 0.03 a	0.53 ± 0.02 b	1.24 ± 0.04 c
procyanidin B2	n.d.	0.48 ± 0.00 a	0.79 ± 0.05 b	1.10 ± 0.04 c	1.42 ± 0.03 d
(−)epicatechin	n.d.	0.28 ± 0.00 a	0.27 ± 0.03 a	0.30 ± 0.02 a	0.31 ± 0.04 a
Dihydrochalcones (mg/100 g d.m.)					
phloretin-2-*O*-xylosylglucoside	n.d.	0.28 ± 0.00 a	0.37 ± 0.00 b	0.60 ± 0.03 c	0.73 ± 0.07 d
phloretin 2-*O*-glucoside (phloridzin)	n.d.	3.29 ± 0.04 a	4.16 ± 0.09 b	6.07 ± 0.05 c	8.16 ± 0.06 d

* Sample coding: see Table 1; different letters in the row denote statistically different average values (α = 0.05, *n* = 2).

**Table 4:** Nutritional value of gluten-free cookies with apple pomace (*n* = 2).

Compounds	Control *	B 15AP	B 30AP	B 45AP	B 60AP
Moisture	6.13 ± 0.10 c	5.46 ± 0.32 b	4.97 ± 0.21 a	4.9 ± 0.37 a	5.02 ± 0.15 a
Protein (g/100 g d.m.)	7.45 ± 0.03 e	6.97 ± 0.01 d	6.48 ± 0.03 c	6.07 ± 0.06 b	5.78 ± 0.01 a
Fat (g/100 g d.m.)	31.70 ± 0.17 a	32.20 ± 0.35 a	32.22 ± 0.27 a	32.90 ± 0.10 b	34.25 ± 0.24 c
Ash (g/100 g d.m.)	0.36 ± 0.01 a	0.41 ± 0.01 b	0.46 ± 0.01 c	0.53 ± 0.01 d	0.57 ± 0.01 e
Available carbohydrates (g/100 g d.m.)	58.55 ± 0.21 e	51.6 ± 0.37 d	46.23 ± 0.31 c	39.8 ± 0.17 b	31.7 ± 0.26 a
Insoluble dietary fiber	0.63 ± 0.05 a	5.68 ± 0.02 b	9.78 ± 0.05 c	14.62 ± 0.01 d	19.17 ± 0.04 e
Soluble dietary fiber	1.31 ± 0.02 a	3.21 ± 0.05 b	4.83 ± 0.06 c	6.09 ± 0.01 d	8.03 ± 0.03 e
Total dietary fiber	1.94 ± 0.02 a	8.83 ± 0.04 b	14.61 ± 0.01 c	20.71 ± 0.01 d	27.71 ± 0.01 e

* Sample coding: see Table 1, different letters in the rows denote statistically different average values (α = 0.05, *n* = 2).

**Table 5:** Texture, volume, and color parameters in gluten-free cookies with apple pomace.

Samples	Hardness (N)		Volume (cm^3^/ 100 g)	L*	a*	b*	Δ*E*
	Day 1	Day 30					
Control ^1^	4.00 ± 0.48 a ^2^	4.10 ± 0.32 a	111.27 ± 1.32 c	75.86 ± 0.36 e	10.01 ± 0.41 a	51.29 ± 0.40 e	16.56 ± 0.16 e
B 15AP	5.08 ± 0.40 b	5.87 ± 0.25 b	106.34 ± 1.76 b	51.33 ± 0.39 d	11.97 ± 0.49 d	29.40 ± 0.62 d	13.62 ± 0.07 d
B 30AP	5.51 ± 0.16 c	6.71 ± 0.68 c	105.94 ± 0.92 b	42.45 ± 0.42 c	11.72 ± 0.14 cd	22.24 ± 0.35 c	12.36 ± 0.14 c
B 45AP	6.35 ± 0.35 d	7.57 ± 3.60 d	104.12 ± 1.33 a	38.02 ± 1.29 b	11.36 ± 0.27 bc	18.40 ± 1.30 b	11.64 ± 0.12 b
B 60AP	8.66 ± 1.20 e	9.45 ± 3.68 e	105.16 ± 0.82 ab	35.74 ± 0.43 a	11.07 ± 0.26 b	16.65 ± 0.56 a	11.27 ± 0.09 a

^1^ Sample coding: see Table 1; ^2^ different letters in the column denote statistically different average values (α = 0.05, for hardness *n* = 7, for volume *n* = 2, for color parameters *n* = 5).

## Data Availability

Not applicable.

## Supplementary Materials

The following supporting information can be downloaded at: https://www.mdpi.com/article/10.3390/antiox12020324/s1, Table S1: Identification of polyphenolic compounds in apple pomace using LC-MS Q TOF; Table S2: Identification of polyphenolic compounds in gluten-free cookies with apple pomace using LC-MS Q TOF).
