# Supplementary material for: Phenolic Compounds and Antioxidant Status of Cookies Supplemented with Apple Pomace
Source: Antioxidants (Basel). 2023 Jan 30;12(2):324. doi: 10.3390/antiox12020324 (PMC9952554; doi:10.3390/antiox12020324)
Supplement: Supplementary file 1 [file antioxidants-12-00324-s001.zip › antioxidants-2139133-supplementary.pdf]

Table S1. Identification of polyphenolic compounds in apple pomace using LC-MS Q TOF

Group of compounds	Name	Rt (min)	λmax [nm]	MS [H-M]-	MS/MS fragments (m/z)
Flavonols	quercetin-*O*-rutinoside	6.85	350	609	301
	quercetin -3-*O*-galactoside	7.02	355	463	301
	quercetin -3-*O*-glucoside	7.13	350	463	301
	quercetin -3-*O*-arabinoside	7.43	355	433	301
	quercetin -3-*O*-xyloside	7.75	350	433	301
	quercetin -3-*O*-rhamnoside	7.90	345	447	301
	isorhamnetin-3-*O*-galactoside	8.59	350	477	315
	isorhamnetin-3-*O*-glucoside	8.82	350	477	315
Phenolic acids	chlorogenic acid	4.37	323	353	191
	cryptochlorogenic acid	4.53	320	353	191
	p-coumaroylquinic acid	5.35	314	337	191
Flavan-3-ols	(+) catechin	4.27	280	289	245
	procyanidin B2	4.94	275	577	289
	(-)epicatechin	5.26	280	289	245
Dihydrochalcones	phloretin-2-*O*-xylosylglucoside	7.60	285	567	273
	phloretin 2-*O*-glucoside (phloridzin)	8.50	285	435	273

Table S2. Identification of polyphenolic compounds in gluten-free cookies with apple pomace using LC-MS Q TOF

Compounds	Rt (min)	λmax [nm]	MS [H-M]-	MS/MS fragments (m/z)
**Flavonols**				
isorhamnetin-3-*O*-galactoside	8.59	350	477	315
isorhamnetin-3-*O*-glucoside	8.82	350	477	315
quercetin-*O*-rutinoside	6.85	350	609	301
quercetin -3-*O*-galactoside	7.02	355	463	301
quercetin -3-*O*-glucoside	7.13	350	463	301
quercetin -3-*O*-arabinoside	7.43	355	433	301
quercetin -3-*O*-xyloside	7.75	350	433	301
quercetin -3-*O*-rhamnoside	7.90	345	447	301
luteolin 6-*C*-hexoside-*O*-hexoside	4.12	340	609	285
luteolin *O*- hexoside-*C*-hexoside	6.59	340	609	285
**Phenolic acids**				
chlorogenic acid	4.37	323	353	191
cryptochlorogenic acid	4.53	320	353	191
caffeoylquinic acid	5.01	320	353	191

p-coumaroylquinic acid	5.35	314	337	191
caffeoyl-dihydroxyphenyllactaoyl-tartaric acid	5.37	320	491	175
2-*O*-*p* -coumaroylglicerol	5.65	314	337	191
1-*O*-*p*-coumaroylglicerol	6.00	314	237	145
p-coumaroylspermidin	6.12	314	237	145
di-*p*-coumaroylspermidin	6.20	314	290	119
Ferullyquinic acid	6.46	325	436	290
**Flavan-3-ols**				
(+) catechin	4.27	280	289	245
procyanidin B2	4.94	275	577	289
(-)epicatechin	5.26	280	289	245
**Dihydrochalcones**				
phloretin-2-*O*-xylosylglucoside	7.60	285	567	273
phloretin 2-*O*-glucoside (phloridzin)	8.50	285	435	273
